# Miscellany

**Published:** 1912-06

**Authors:** 


### MISCELLANY

### Coffee Imports of the United States and Share of World's Consumption

More than one-third of the 2 1-2 billion pounds of coffee annually entering the international commerce of the world is consumed in the United States, its imports of that article being twice as much as those of Germany, three times those of Netherlands, four times those of France, nearly ten times those of Great Britain, and half as much as those of all Europe, next to the United States the great coffee-consuming section of the world.

The world's leading importers of coffee, according to the latest official reports of the various countries thus far received by the Bureau of Statistics, Department of Commerce and Labor, are: the United States, 875 million pounds; Germany, 404 million; Netherlands, 265 million; France, 245 million; Austria-Hungary, 127 million; Belgium, 95 million; the United Kingdom, 88 million; and Sweden, 65 million. Italy, Norway, Switzerland and Denmark also consume considerable quantities, ranging from 45 to 25 million pounds each. Of the countries on the western hemisphere, Argentine and Cuba each import about 20 million pounds per annum; Canada, about 8 million; Chile, 7 million, and Uruguay about 3 million, while Australia, the island continent, consumes between 1 1-2 and 2 million pounds per annum. Certain of the African countries are comparatively large importers of coffee, the imports into Egypt averaging about 15 million pounds a year; the Cape of Good Hope, 20 million, and Algeria, about 15 million pounds.

While the United States is the world's largest consumer of coffee, the imports have not increased during recent years. In the fiscal year 1902, for example, the imports aggregated 1,091 million pounds; in 1905, 1,048 million; in 1909, 1,050 million; in 1911, 875 million; and in the present year will probably aggregate approximately 800 million pounds, or considerably less than the annual average of the period since 1900. In 1871 imports of coffee amounted to 318 million pounds; in 1881, 455 million; in 1891, 520 million; in 1901, 855 million; and in 1911, 875 million. Cocoa has to a large extent supplanted coffee as an American beverage, the imports having increased from 3 1-2 million pounds in 1871 to over 140 million pounds in 1911.

